# Comparative life cycle assessment for the manufacture of bio-detergents

**DOI:** 10.1007/s11356-022-24439-x

**Published:** 2022-12-12

**Authors:** Javier Mauricio Villota-Paz, José Luis Osorio-Tejada, Tito Morales-Pinzón

**Affiliations:** 1 Faculty of Environmental Sciences, Universidad Tecnológica de Pereira, Alamos, Pereira, Colombia; 2 Faculty of Engineering, Universidad Mariana, Pasto, Colombia; 3 School of Engineering, University of Warwick, Coventry, UK

**Keywords:** LCA, Detergents, Soap, Cleaning, Washing, Sustainability, Bioproducts

## Abstract

The increasing consumption of cleaning products deteriorates water resources due to harmful components such as phosphorus (P) and nitrogen (N) compounds, oils, bleach, and acids, typical compounds in traditional detergents. The use of biodegradable detergents as an environmentally friendly alternative has been proposed in different regions. In Colombia, resolution 1770/2018 sets a minimum biodegradability rate of 60% for the surfactants present in liquid detergents, which would reduce to a similar extent the impacts on water after their use. However, the environmental impacts of the supply chain of these detergents and their raw materials have not been evaluated so far. This study presents an environmental life cycle assessment of petroleum-based liquid detergents and a comparison to traditional solid detergents, based on the ISO 14040 standard and the ReCiPe-2016 impacts assessment method. A novel bio-detergent containing anionic plant-based surfactants was proposed in this analysis. The impacts of packaging and the distribution of the product to consumers were also considered. Raw materials contributed to 91% of the total of 314 g of CO_2_ eq generated per liter of liquid detergent, where the production of fatty alcohol sulfate and PET packaging shared 78.8% and 12.2% of the total impact, respectively. It was also determined that 5.4 L of water are consumed and 0.09 g of P eq and 0.1 g of N eq are emitted per liter of detergent. This liquid detergent presented better environmental performance than traditional detergents in all the impact categories, except for the fossil resource scarcity category. The evaluated detergent would significantly mitigate the generation of negative effects on ecosystems. Moreover, the substitution of PET for HDPE packaging could reduce the impacts on freshwater eutrophication by 10%, although the carbon footprint can slightly increase, which could be compensated due to its higher recyclability rate. In contrast, the proposed bio-detergent would not have significant benefits and would negatively affect water consumption and land use in its supply chain.

**Supplementary Information:**

The online version contains supplementary material available at 10.1007/s11356-022-24439-x.

## Introduction

Nowadays, there is a great variety of substances used for washing clothes and cleaning dirt from different surfaces (Bianchetti et al. 2015). These substances or detergents are composed of mixtures of surfactants, bleaching agents, enzymes, and other minor additives (Smulders et al. 2012; Cheng et al. 2020). The increasing usage of detergents is concerning since it could negatively affect the environment and human health (Giagnorio et al. 2017). However, detergents are still part of everyday use in human life for hygienic and cleaning purposes (Wang et al. 2019).

Solid soaps and traditional detergents harm the environment due to emissions of polluting compounds, such as the formation of non-biological chemical particles that mainly affect water sources and generate greenhouse gas (GHG) emissions. Raw materials in detergents generate carbon emissions due to the use of complex chemical compounds (Golsteijn et al. 2015). Moreover, it has been identified that the presence of surfactants in solid detergents causes toxicity in water sources and affects marine life (Rebello et al. 2019; Mousavi and Khodadoost 2019). Likewise, the deterioration of water ecosystems is due to the emissions of anionic surfactant, non-anionic, pure anionic components, and chemical elements such as sodium (Na), which are not environmentally friendly, generating pH alterations and contributing to the eutrophication of water bodies (Falbe 1987).

As an alternative to the environmental impacts caused by traditional detergents, which are characterized by containing in their chemical structure synthetic elements, such as surfactants (Solé Cabanes 2014), different sustainability alternatives associated with the production of detergent products from organic sources have been proposed, promoting the development of green products that involve simpler chemical structures to increase biodegradability and thereby mitigate environmental impacts (Arthur et al. 2012). In Colombia, resolution  1770/2018 (Colombian Government 2018) defines biodegradability as the susceptibility of a chemical compound or substance to being decomposed after interactions with microorganisms, specifically in the presence of oxygen, generating carbon dioxide, water, and mineral salts.

The use of anionic surfactants from natural raw materials such as plant-based oils to manufacture liquid detergents can increase their biodegradability rate and reduce the use of petroleum-based materials. In this sense, these plant-based cleaning products could be considered bio-detergents.

Regarding the development of this alternative, the use of plant-based oils to replace a fraction of the petroleum-based surfactants has been implemented, which come from plants such as coconut, oil palm, canola, and others, whose cleaning properties similar to those of traditional detergents have been observed (Do et al. 2019). Some studies revealed that palm oil (*Elaeis guineensis*) can be considered a potential natural surfactant, which can offer consumers confidence as a functional product (Saxena et al. 2017; Slamet et al. 2017). However, the formulation of a bio-detergent suggests a paradigm in terms of quantification of environmental impacts, which might arise in its production process (Javed and Qazi 2016; Singh et al. 2019).

To estimate and evaluate the environmental impact that can be generated by the manufacturing of a bio-detergent compared to a traditional detergent, it is necessary to perform a life cycle assessment (LCA) of each of the products, using tools capable of quantifying the impacts in each of the stages of this process. In the Colombian market, different national and international brands offer liquid and solid detergents free of toxic components, which would not generate harmful effects on health in case of ingestion. Likewise, eco-friendly detergents seek to attract different types of consumers and generate in them a sense of conformity toward the responsible use of natural resources (De Koning et al. 2010). Because of this premise, it is necessary to analyze whether there is a product that can meet different eco-design conditions to reduce the generation of negative environmental impacts (Balboa et al. 2014).

The objective of this study is to perform a critical analysis and compare the environmental performance of the life cycle of liquid detergents in comparison to a traditional detergent (TD). These detergents are used for washing clothes and different surfaces. The potential environmental impacts of each of the products were estimated, and improvement strategies were identified to determine which product and its supply chain have better environmental performance.

## Materials and methods

This research was conducted at the production plant of Probionar S.A.S., a company located in the municipality of Buesaco at 2859 m above sea level, in Nariño, Colombia. This plant was created in 2015 with resources from the Emprender Fund, for the manufacture and marketing of liquid detergents under a sustainable business model. The LCA methodology described by the ISO 14040 standard (ISO, 2006) was applied. This study includes four phases: goal and scope of the study, life cycle inventory (LCI) analysis, life cycle impact assessment (LCIA), and interpretation.

### Goal and scope of the study

The goal of the study was to benchmark and identify the critical inputs in the supply chain of liquid detergents produced by Probionar against TD, in a system boundary from cradle-to-site (Osorio-Tejada 2022). This boundary considers the impacts of obtaining raw materials and the transformation of the product up to the factory gate (cradle-to-gate), plus the impacts of the distribution of the product to its site of use (gate-to-site).

The main product of the company is bio-multipurpose liquid detergent (BLD). BLD can be used for washing clothes, floors, plastic parts, furniture, rugs, and kitchen equipment. The prefix “bio” in the name of the product was included by the company because it was formulated to have high and fast biodegradability characteristics. BLD contains only 9.7% w/w of sodium lauryl ether sulfonate surfactant (fatty alcohol sulfate), a lower concentration than other similar products on the market that can contain up to 20% w/w. This surfactant stands out due to its higher biodegradation rate (Co: 0.5 mg/L → 0.4854 day^−1^, Co: 5 mg/L → 0.4725 day^−1^, Co: 10 mg/L → 0.413 day^−1^), which is in the margin of 15 days (> 90%). It was shown that at low concentrations of surfactants, the biodegradation kinetics is better because the microorganism does not become unstable (Herrera Torreblanca 2017). It is mentioned in this same study that, theoretically, all surfactants are biodegradable with time and appropriate conditions, but it will depend on the action of microorganisms that solve the problem of contamination caused by substances, which must degrade easily and in a short time.

From the holistic perspective, the aim is to establish a more precise contextualization of the phases or processes that are present in the entire product system, obtaining, as a result, a direct estimate that is associated with the environmental impacts generated by each of the detergents (Bjørn et al. 2017).

The functional unit for this analysis was 1 L of BLD, the best-selling presentation packaged in a polyethylene terephthalate (PET) bottle. The performance of the main product, BLD, is 20 laundry washes per liter of product. That is, to wash a load of 12 garments (5 kg of clothes) a total of 49.92 g of product are needed, considering a density of 1.03 g/mL BLD. The cut-off approach for the LCA study was selected under the hierarchical perspective. This is a consensus method and one of the most widely used in LCA studies concerning the consumption of detergents in the laundry industry (Giagnorio et al. 2017).

With the aim of reducing dependence on fossil sources, Probionar S.A.S has introduced a novel bio-detergent containing anionic plant-based surfactants, which has also been demonstrated to improve the biodegradability period. Moreover, the use of HDPE instead of PET containers to improve reuse rates due to their higher durability was analyzed.

The study focused on the analysis of midpoint results or intermediate environmental impact categories, which are closer to the environmental intervention (Vallejo 2004). The ReCiPe 2016 method was used to analyze the potential impacts on climate change, freshwater and marine eutrophication, freshwater and marine ecotoxicity, land use, scarcity of fossil resources, and water consumption. These midpoint results were aggregated into endpoint indicators, which express the relative severity of the effects on human health, ecosystems, and resources (Huijbregts et al. 2017).

### Life cycle inventory analysis

Data were collected from primary sources for the transformation processes of BLD and its equivalent TD, as well as for vehicle types and distances for product distribution. In addition, primary sources were also used for the identification of the distances traveled by the raw materials used in the transformation processes. The major components of TD are sodium hydroxide, palm oil, and water.

In this sense, the default datasets for the raw materials of the Ecoinvent version 3.6 database (ETH, 2022) were used and managed with SimaPro v9 (PRé Consultants, 2022). Material flows were adapted to the conditions of the local territory used for its manufacture and transport, e.g., specific Colombian electricity mix, natural and drinking water, and the regulated diesel B10 (10% v/v biodiesel content). The specific LCI datasets for the BLD and TD, as well as detailed information about the origin of raw materials, suppliers, distance, and type of vehicle, are presented in Tables S1 to S4 in Supplementary Material SM1. The technical datasheet for the biodegradability rate of the plant-based BLD, elaborated by an accredited laboratory via the OECD 301F standard, is presented in Supplementary Material SM2.

## Results and discussion

### Impact assessment: midpoint analysis

**Fig. 1:**
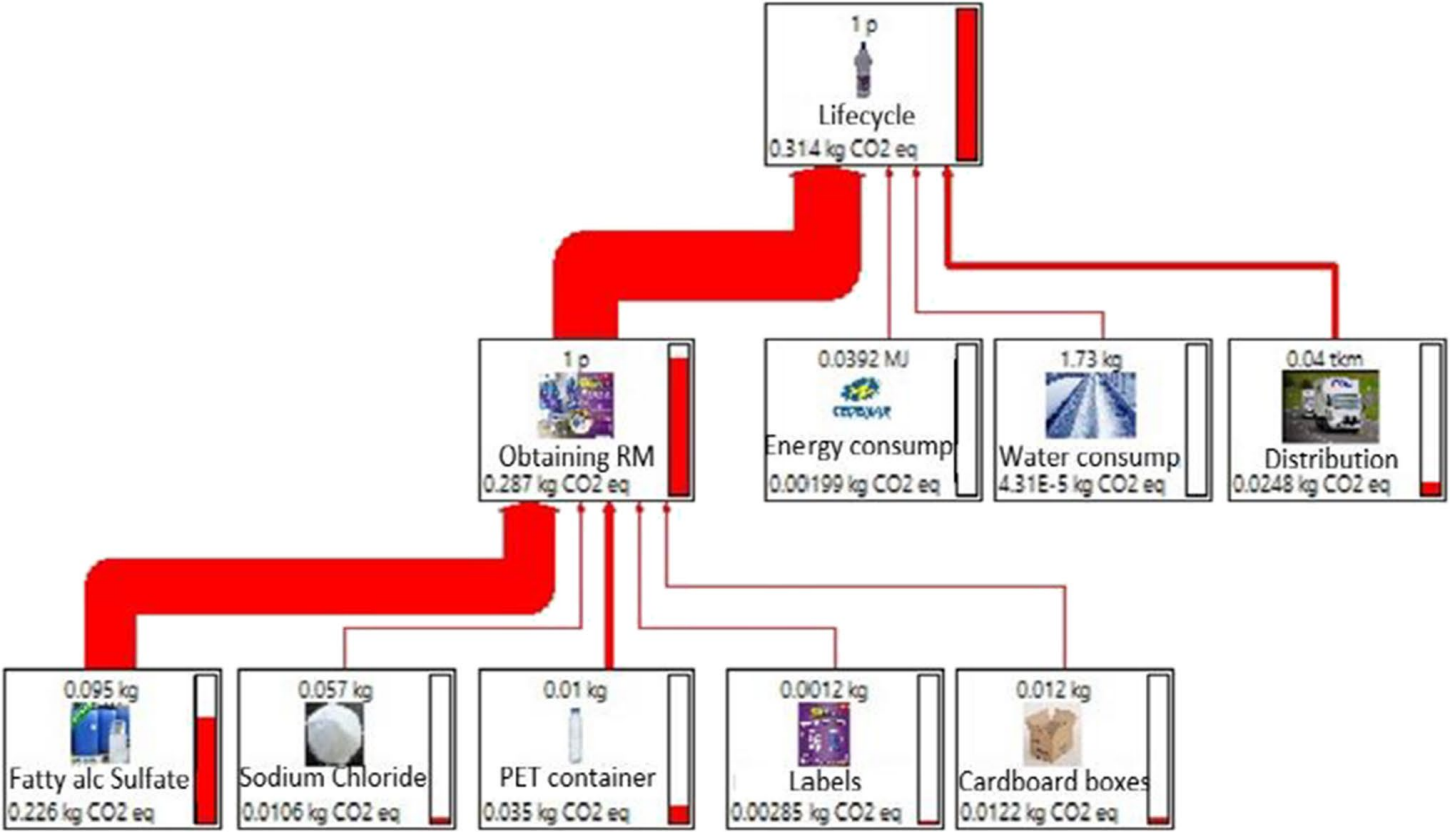
Cradle-to-site assessment for 1 L of BLD—climate change. Note: bio-detergent considering petrochemical surfactant and PET container. This image was obtained from SimaPro software

The analysis of the life cycle of the product allowed us to show the network diagram of each of the impacts evaluated based on the three stages defined by the limits of the system. Figure 1 shows the results on climate change, which considers the results of the integrated phases of procurement, transformation, and distribution of raw materials. The procurement of raw materials is the main contributor to total carbon emissions. The greatest environmental impact at this stage was due to the consumption of surfactants (fatty alcohol sulfate), which contributes to 78.8% of the emissions of the raw materials stage.

**Fig. 2:**
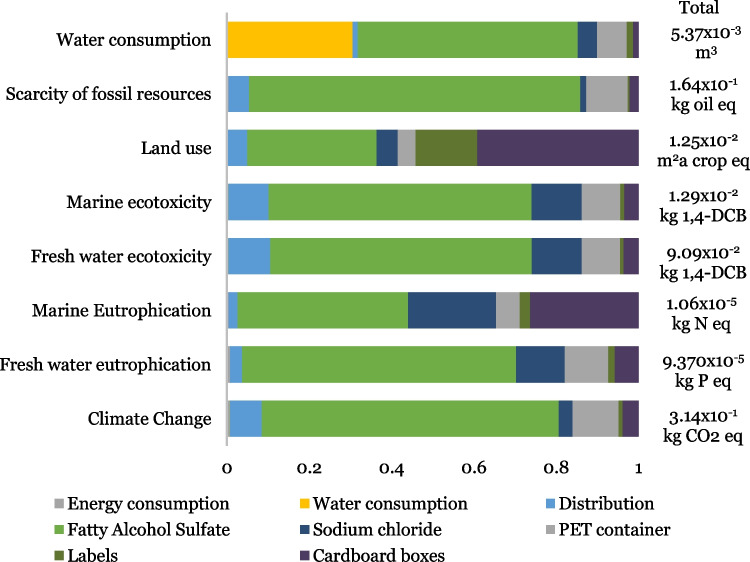
Cradle-to-site assessment for 1 L of BLD—midpoint impact categories

To analyze the midpoint impacts generated in the life cycle of the product, each of the eight environmental impact categories mentioned above is considered for the three stages required to produce 1 L of BLD as shown in Fig. 2. The raw materials stage includes data on fatty alcohol sulfate, sodium chloride, PET containers, labels, and cardboard boxes. The transformation stage uses data on energy consumption and water consumption.

From Fig. 2 for BLD production, we can state that the greatest contribution in all impact categories is due to raw materials procurement. It can also be identified that this stage had the most significant impact on climate change with 0.287 kg of CO_2_ eq, followed by the scarcity of fossil resources with 0.156 kg oil eq, representing 94.5% and 94.6% of the total life cycle emissions, respectively.

### Impact assessment: endpoint analysis

**Fig. 3:**
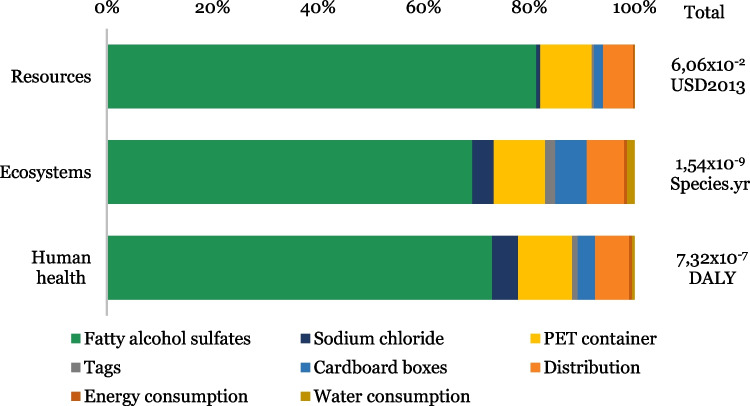
Evaluation of environmental impacts by endpoint impact categories

In order to make a comparison between the impacts generated at a midpoint and endpoint level, the potential impacts on human health, ecosystems, and resources are presented in Fig. 3.

In Fig. 3, it can be seen that to produce 1 L of BLD, the stage of raw materials procurement is the most relevant. Inputs such as fatty alcohol sulfate, PET packaging, and sodium chloride generate potential negative effects in the different areas of protection. Fatty alcohol sulfates generate the highest impacts with contributions of about 73% (5.34 × 10^−7^ DALY), 69% (1.07 × 10^−9^ species.year), and 81% (0.0493 USD2013), on human health, ecosystems, and fossil resources, respectively.

The analysis of critical points made it possible to identify that the inputs with the greatest impact on the raw material production stage are the surfactants. Although it is currently found in the literature that most modern surfactants are easily biodegradable and have low toxicity in the aquatic environment, most of them are synthesized using petroleum (Kjellin and Johansson 2010). In the particular case of the product under study, the surfactant called “fatty alcohol sulfate” was used, which has a wide application in industry, as anionic, cationic, and nonionic surfactants, used as detergents, which have between 12 and 18 carbon chains (Ríos et al. 2006). Hence, the consumption of petroleum-derived surfactants importantly affects the scarcity of fossil resources category.

### Comparison between BLD and TD

BLD and TD can be used for washing clothes or textile garments. To wash a load of 5 kg of clothes, 49.92 g of BLD or 99.99 g of TD is required. In other words, to clean the same amount of laundry washed with a liter of BLD, it is necessary to use about 2 kg of solid TD. These values were obtained through performance tests. The inventory dataset for the TD is presented in Table S4 of the Supplementary Material file.

**Fig. 4:**
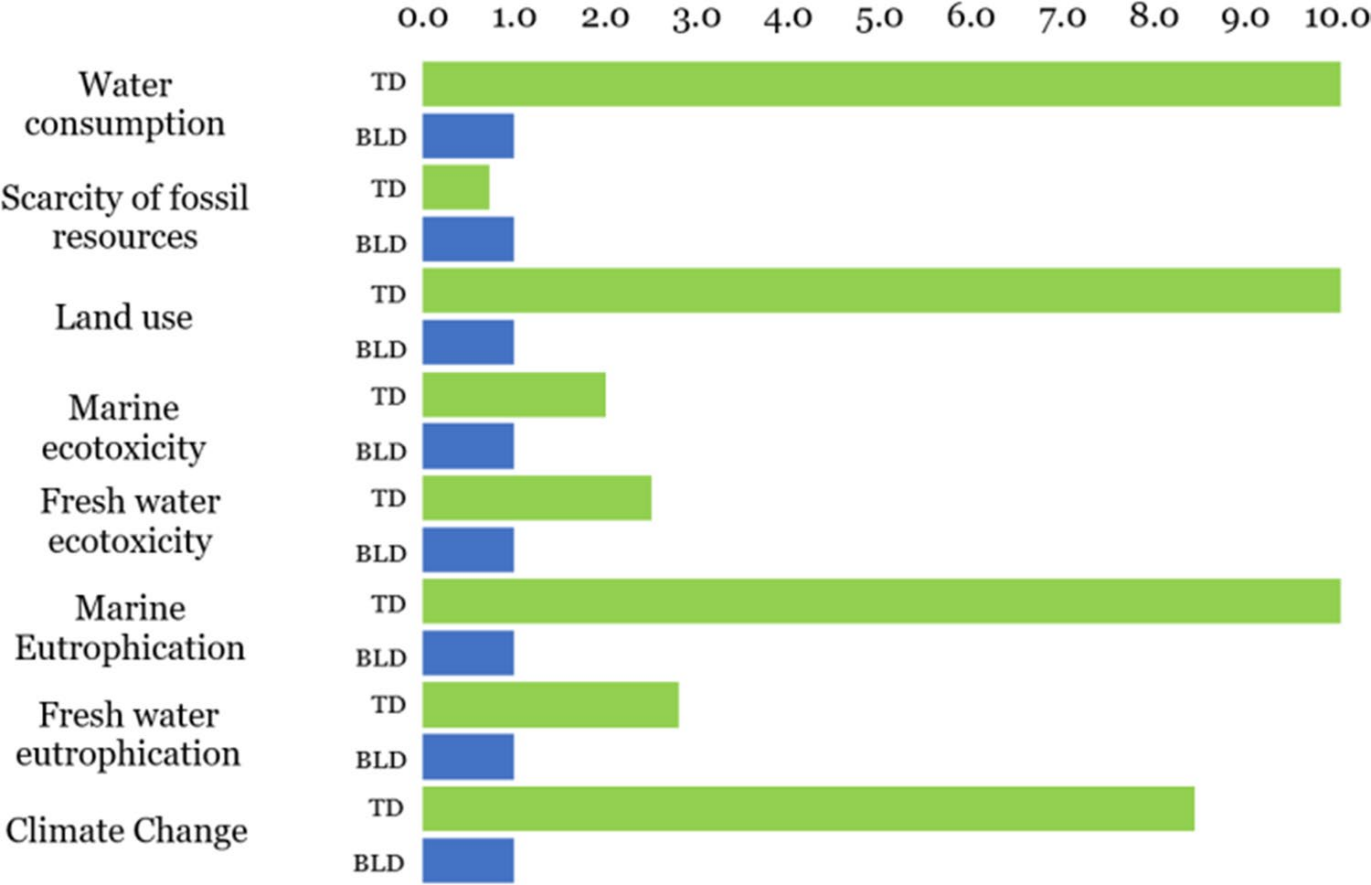
Midpoints LCA comparison—TD impacts variation in relation to the BLD impacts; the latter normalized to 1.0

Figure 4 shows that BLD has an advantage as it represents a reduction in TD-related environmental impacts, which contributes to mitigating the impacts generated by emissions on aquatic ecosystems. The categories where the BLD shows the greatest reduction in impacts are land use and marine eutrophication. In addition, the impacts of BLD on climate change and water consumption are 88% and 91% lower than those generated by solid TD.

In Fig. 4, the impacts of TD can be up to 10 times higher than the impacts of BLD. This implies that low cleaning performance is not the only factor generating environmental impacts. Most of the impacts of TD are due to the oil source required for the saponification process with sodium hydroxide. Comparatively, 4.5 times more oil source is needed to produce one kg of TD than is needed to produce 1 L of BLD. This explains the higher impacts of TD in most of the categories. The exception of the low impacts of TD in the category of scarcity of fossil resources was because only vegetable oils were used for the saponification process, while petroleum-derived surfactants were used for BLD.

### Interpretation

The LCA of BLD showed that 1 L of this product generates a total of 0.314 kg of CO_2_. It is worth mentioning that there are some impacts in the consumption and post-consumption phase, which were not considered, and they would surely increase this value.

The life cycle water consumption to produce 1 L of the product was 5.4 L, of which the surfactant consumed 1.6 L, being the input that most affects the environment. It should be noted that, although water consumption is not high in comparison with other products, it is advisable to propose strategies to reduce this resource used to obtain the detergent.

Another category that requires careful analysis and should be considered is the eutrophication of fresh and marine water because phosphorous and nitrogen are involved in the production of detergents. The results obtained were 0.09 g of P eq and 0.1 g of N eq per liter of BLD, which are relatively low for a detergent (Madariaga et al. 2007).

According to the findings, it was recommended to Probionar S.A.S. to evaluate the feasibility of substituting the surfactant “fatty alcohol sulfate”, which, although it is low in phosphorus and nitrogen, is a petroleum derivative. Concerning the above, the implementation of a surfactant that is not derived from petroleum was proposed, which would mitigate some environmental impacts associated with the scarcity and thus the dependence on petroleum.

Another recommendation for the company was associated with the substitution of the PET-type container for an HDPE-type container, which would allow the container to be reused more times. From this action, the company must comply with a 30% return of this plastic until 2030 according to resolution 1407/2018 (Colombian Government, 2018).

Therefore, a sensitivity analysis for the impacts of the implementation of these recommendations is performed in sections “Comparison between PET and HDPE container” and “Discussion”. 

### Comparison between petrochemical and plant-based surfactants

**Fig. 5:**
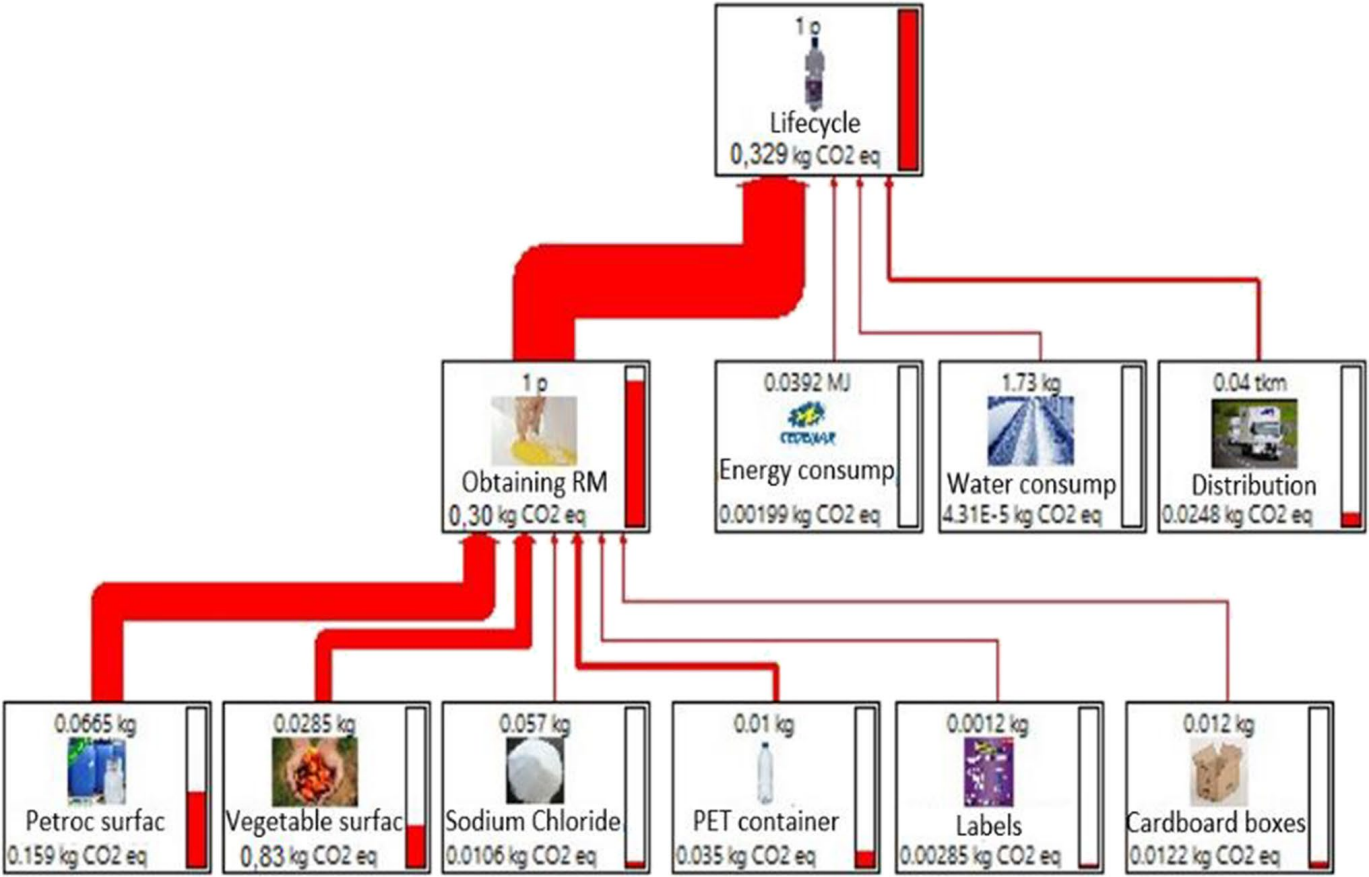
Cradle-to-site assessment for 1 L of BLD (30% plant-based surfactant)—climate change. Note: bio-detergent considering 70% petrochemical- and 30% plant-based surfactant and PET container. Note: This image was obtained from SimaPro software

According to the data provided by the supplier of the plant-based surfactant, it contains 30% palm oil and 70% surfactant of petrochemical origin. Probionar S.A.S has used palm oil to produce the plant-based surfactant because it is the most cost-effective vegetable oil for industrial applications in Colombia. Using this new surfactant, the impacts on climate change increase from 0.314 to 0.329 kg CO_2_ eq per liter of BLD, as shown in Fig. 5. The inventory dataset for the BLD with plant-based surfactant is presented in Table S3 of the Supplementary Material file.

For this analysis, the dataset of the life cycle of palm oil in Colombia was used including the distances traveled from the plantation and refinery to its reception at the plant (Gómez and Gustavo 2015; Osorio-Tejada et al. 2018; Pertuz Martínez and Santamaría Escobar 2014). In the production of the surfactant using palm oil, the sulfates of fatty alcohols are obtained by sulfonation of the alcohols, and a thin film of alcohol is bound with a mixture of sulfur oxide (SO_3_) and dry air inside a vertical tube. After sulfonation, sodium hydroxide is applied to neutralize the acid that has been formed.

**Fig. 6:**
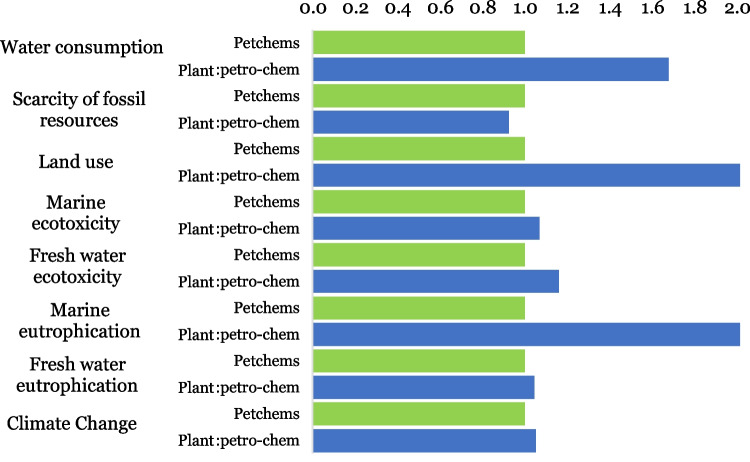
Midpoints LCA comparison—plant-based surfactant results variation in relation to the petrochemical-based surfactant results; the latter normalized to 1.0

To identify which surfactant turns out to be more environmentally friendly, the midpoint results between the conventional and the new proposed formula are presented in Fig. 6.

From Fig. 6 it was possible to interpret that the new product with a mixture of plant- and fossil-based surfactants does not contribute to reducing environmental impacts in seven of the eight categories studied. Only in the category of the scarcity of fossil resources, impacts were reduced by 8%.

### Comparison between PET and HDPE container

**Fig. 7:**
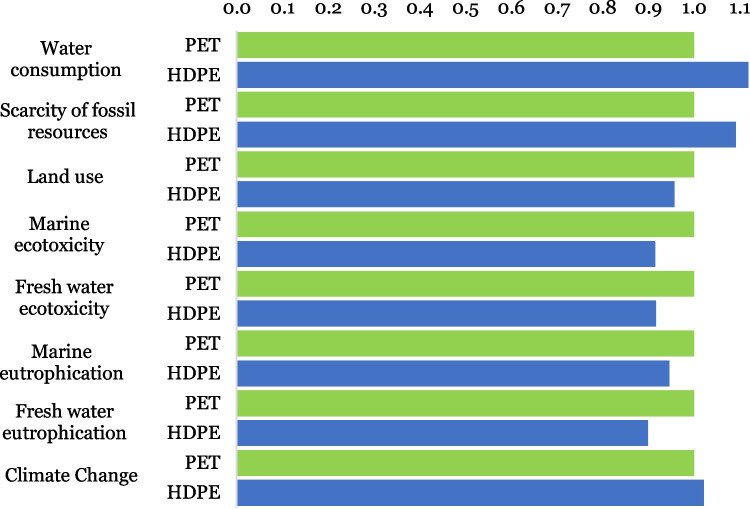
Midpoints LCA comparison—HPDE impacts variation in relation to the PET impacts; the latter normalized to 1.0

A comparison was made between the two types of containers that the company can use to package a liter of BLD. Based on the data provided by the company for a yearly production of 5323 L of BLD, 60.34 kg of PET-type packaging was used, while for HDPE packaging, 117.2 kg must be used, i.e., 0.011 kg or 0.022 kg per liter of BLD, respectively. The inventory dataset for 1 L of BLD using petrochemical surfactant and HDPE container is presented in Table S2 of the Supplementary Material file. It should be noted that the geographical scope of this study focuses on the department of Nariño, Colombia. The cradle-to-site assessment results of 1 L of BLD using PET and HDPE containers are presented in Fig. 7.

From Fig. 7, we can conclude that the use of HDPE is beneficial in most of the impact categories. The greatest impact reduction due to the use of HDPE compared to PET packaging corresponds to freshwater eutrophication with a 10% reduction. Likewise, in the categories of ecotoxicity of fresh and marine water, the use of HDPE reduces the impacts by 8%. It should also be considered that most plastic packaging has petroleum-based polymers in its composition, which can accumulate in ecosystems for long periods (Zhao et al. 2020). One of the most adverse factors for the environment is the final disposal of PET containers because this waste accumulates in ecosystems, reducing the useful life of sanitary landfills, and when PET containers are incinerated, it can generate greenhouse gases, affect human health, and the ashes contaminate groundwater through leachate infiltration (Colombian Government 2004). The advantage of HDPE containers lies in the fact that they can be more easily recycled, as well as they are characterized by their long useful life as a functional product and by recovering high concentrations of the HDPE material in their reuse process (Grigore 2017; Viva 2013). Therefore, a campaign is proposed to promote recycling because only 26% of the total plastic is recycled in Colombia, which would significantly contribute to the reincorporation of these materials in the production cycle, in addition to mitigating the environmental impact generated by the accumulation of plastics (Carvajal and Salas 2018).

### Discussion

Based on the data found in the product life cycle analysis, it was possible to identify that the variations in the initial system generate representative changes, for which the system is considered sensitive.

The traditional detergent is only more environmentally friendly than the presented BLD in the category scarcity of fossil resources (see Fig. 4).

According to the data presented in Fig. 7, it can be stated that PET packaging favors the categories of eutrophication of fresh and marine water, ecotoxicity of fresh and marine water, and land use. But at the same time, it increases the carbon footprint of the product by 7 g of CO_2 eq_, the water footprint by 0.6 L, and oil consumption by 15 g of CO_2 eq_. The substitution of PET for HDPE packaging indicated that this action benefits five of the eight impact categories; although the carbon footprint is increased, the negative effects of this category might be reduced thanks to the plastic packaging recovery campaign.

On the other hand, the only category that benefited from the substitution of 30% of fossil-based surfactant for a plant origin (palm oil) was the scarcity of fossil resources. This strategy reduced the consumption by 12.5 g of oil equivalent per liter of BLD, which is an annual reduction of 66.5 kg of oil equivalent.

The new surfactant containing 30% palm oil resulted in higher negative impacts in most of the categories because palm oil is mostly produced in locations with low development of sustainable crops. These impacts can be associated, for example, with inadequate land use in palm cultivation, destructive deforestation, and habitat degradation, as well as with some environmentally harmful inputs used in the manufacture of fatty alcohol sulfate, such as solvents, and sodium hydroxide, and sulfur oxide, among others. Half of the palm oil in Colombia has been planted in former pastures or annual crops. However, the fraction planted in rainforest areas generates most of the environmental impacts, as observed worldwide for this type of energy crop, mainly in Southeast Asia. For example, it is found that Indonesia, the largest oil palm producer worldwide with more than 700 extraction mills (Nasution et al. 2018), is the third-largest greenhouse gas emitter in the world (Parlour 2017), mainly due to the rainforest and peatlands destruction. Other studies indicate that the environmental impacts generated palm oil production, in countries such as Thailand, are mainly due to the burning of fibers in boilers, the use of fertilizers, wastewater treatment and disposal of empty fruit bunches, the use of gasoline in motor mowers, and the use of glyphosate for weed control (Saswattecha et al. 2015). These activities in turn are related to specific impacts in different categories, such as climate change, associated with high methane (CH_4_) emissions from wastewater in open ponds in the milling phase, and nitrous oxide (N_2_O) emissions from the application of nitrogen fertilizers in the cultivation phase. Moreover, air pollutants are generated in palm oil production during fuel combustion, such as particulate matter (PM), sulfur dioxide (SO_2_), nitrogen oxides (NOx), non-methane volatile organic compounds (NMVOCs), and carbon monoxide (CO) (Saswattecha et al. 2015). Other categories impacted due to palm oil production are ozone formation, acidification, and eutrophication. The latter is associated with the high use of nitrogen and phosphorus fertilizers used to improve yields in oil palm crops, which cause eutrophication in surface water (Saswattecha et al. 2015). Other options to diversify the use of surfactants in the manufacture of bio-detergents can be found in LCA studies that seek to generate alternatives to meet the growing oil demand for industrial use. According to Schmidt (2010), palm oil is environmentally preferable to rapeseed oil, in some environmental impact categories such as ozone depletion, acidification, eutrophication, photochemical smog, and land use. Meanwhile, rapeseed oil is preferable over palm oil in categories such as climate change and biodiversity.

Therefore, plant-derived surfactants can become a substitute for petrochemicals, provided that the methods of obtaining the plant-derived surfactant are ecologically sustainable.

Regarding the completeness and consistency of the study, for the modeling of the assessed product, real data provided by suppliers, customers, and the company on the flow of materials were used, both qualitatively and quantitatively, to ensure the reliability of the data. The inputs were selected from Ecoinvent 3.6 database and were adjusted for Colombia concerning local water and energy consumption. Likewise, transportation required for the raw materials routes was adapted for the actual distances between the suppliers and the company, using tools such as Google Maps to identify the most accurate and shorter distances, thereby greatly reducing the levels of uncertainty in the study.

#### Consumer awareness campaign

An environmental strategy proposed to decrease the impact of production operations on the natural environment is the adequate use of resources and optimization of energy waste, using the ISO 14001 methodology, which focuses on green sustainability, environmental management systems, and natural resources (Latan et al. 2018). Therefore, taking into account the LCA of BLD, a strategy focused on the final disposal of HDPE packaging was proposed, applying concepts of some sustainable development goals (SDG 12 Responsible Production and Consumption) and thus being able to comply with various environmental management regulations in Colombia.

In this context, the commitment of the company is to reduce the environmental impacts generated in the production of the BLD product. A campaign was conducted to return the HDPE container to the company Probionar S.A.S to give it adequate handling in terms of disinfection and conditioning for reuse. This campaign was called “*Redímete con el Medio Ambiente*” (Redeem yourself with the Environment) and managed to return 36% of the plastic used to supply the canteens of the official educational institutions of the municipality of San Juan de Pasto (Colombia).

**Fig. 8:**
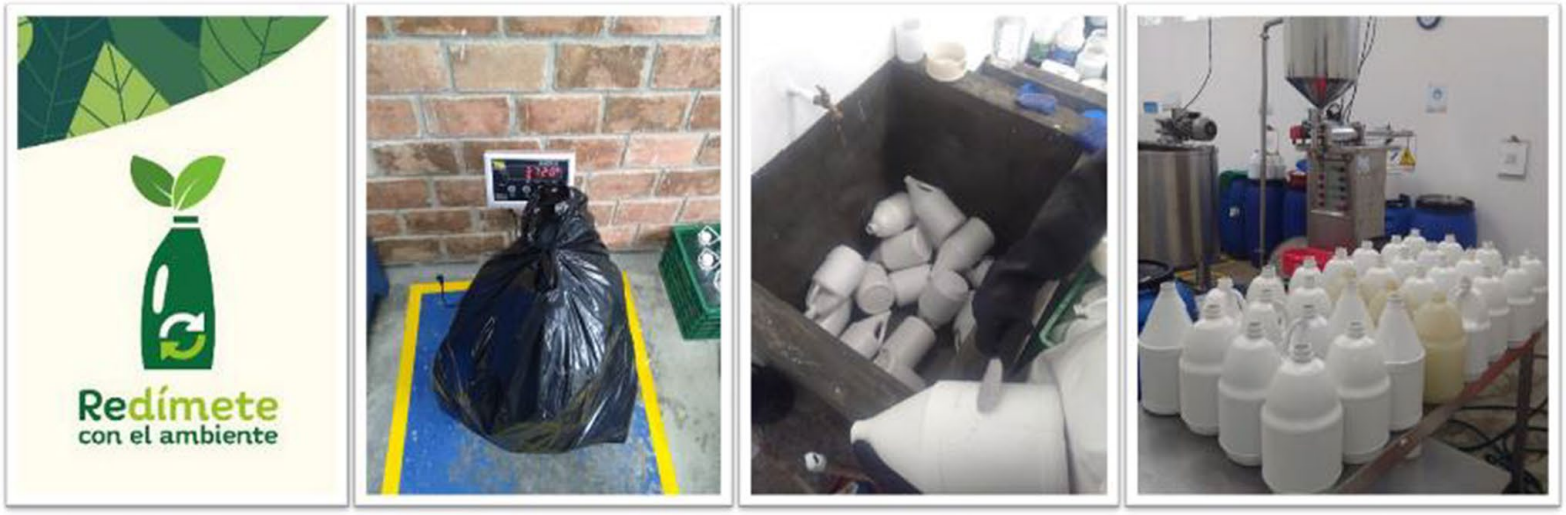
Campaign redeem yourself with the environment

Figure 8 shows photographs of the implemented campaign, which, in addition to bringing benefits to the environment, generates a reduction in manufacturing costs for the company because the recovered packaging is cheaper than virgin packaging. It also generates an incentive for the food handlers or cleaners because by returning these containers to the company, they can obtain points, which can be redeemed for prizes at the end of an academic school term. This campaign was disseminated through the school restaurants of the municipal educational institutions through talks and training for the implementation of the environmental campaign. In addition, a publicity campaign was carried out with information about the alternative proposed by the company to share with customers interested in learning more about this campaign and its benefits for the environment and economy.

## Conclusions 

The study made it possible to calculate specific impacts to produce 1 L of the bio-multipurpose liquid detergent, obtaining a carbon footprint of 0.314 kg of CO_2 eq_, a water footprint of 5.4 L, a phosphorus footprint of 0.09 g of P eq, and a nitrogen footprint of 0.1 g of N eq. Within the three stages of the life cycle studied, the raw material procurement stage contributed to 91% of the impacts on climate change. In this stage, the inputs with the greatest impact were fatty alcohol sulfate (78.8%) and PET packaging (12.2%).

In addition, it was concluded that the use of HDPE packaging would reduce most environmental impacts, but to maximize its benefits, it is recommended that the containers must be reused. On the other hand, the use of a plant-based surfactant does not guarantee that the impacts will be globally reduced; therefore, it is first necessary to know the conditions of palm oil production.

Finally, it was determined that the bio-detergent generates less environmental impact than traditional detergent. The formulation of this liquid bio-detergent contributes greatly to reducing the environmental impact caused by these cleaners on textile garments. In this sense, this analysis also made it possible to show which strategies would be the most effective to further reduce the overall impact of the product and generate competitive advantages for the company.

## Supplementary Information

Below is the link to the electronic supplementary material.

Supplementary file1 (DOCX 30.9 KB)

Supplementary file2 (PDF 788 KB)

## Data Availability

Supporting data are available as electronic supplementary material. Additional information would be provided upon request.

## References

[CR1] Arthur T, Harjani JR, Phan L, Jessop PG, Hodson PV (2012). Effects-driven chemical design: the acute toxicity of CO 2-triggered switchable surfactants to rainbow trout can be predicted from octanol-water partition coefficients. GreenChem.

[CR2] Balboa C, Hermida C, Domínguez M (2014). Circular economy as an ecodesign framework: the ECO III model. Informador Técnico.

[CR3] Bianchetti GO, Devlin CL, Seddon KR (2015). Bleaching systems in domestic laundry detergents: a review. RSC Adv.

[CR4] Bjørn A, Owsianiak M, Laurent A, Olsen SI, Hauschild Corona A, & Z, M (2017) Scope definition. Life cycle assessment: theory and practice10.1007/978-3-319-56475-3

[CR5] Carvajal EAV, Salas GLZ (2018). Conscientious objection to euthanasia: a personalistic bioethical analysis for the Colombian case. Rev Lasallista Investig.

[CR6] Colombian Government (2004) Environmental guide for the plastics sector (in Spanish). Ministry of Environment, Housing and Territorial Development, Bogotá, Colombia

[CR7] Colombian Government (2018) Resolution 1770/2018 (in Spanish). Ministry of Health and Social Protection and Ministry of Environment and Sustainable Development, Bogotá, Colombia

[CR8] Cheng KC, Khoo ZS, Lo NW, Tan WJ, Chemmangattuvalappil NG (2020). Design and performance optimisation of detergent product containing binary mixture of anionic-nonionic surfactants. Heliyon.

[CR9] De Koning A, Schowanek D, Dewaele J, Weisbrod A, Guinée J (2010). Uncertainties in a carbon footprint model for detergents; quantifying the confidence in a comparative result. Int J Life Cycle Assess.

[CR10] Do DN, Dang TT, Le QT, Lam TD, Bach LG, Nguyen DC, Toan TQ (2019). Extraction of saponin from gleditsia peel and applications on natural dishwashing liquid detergent. Mater Today Proc.

[CR11] ETH (2022) Ecoinvent LCA database. Ecoinvent v38 2022. www.ecoinvent.org (accessed May 22, 2022)

[CR12] Falbe J (ed.) (1987) Surfactants in consumer products: Theory, Technology, and Application, Springer-Verlag, New York

[CR13] Giagnorio M, Amelio A, Grüttner H, Tiraferri A (2017). Environmental impacts of detergents and benefits of their recovery in the laundering industry. J Clean Prod.

[CR14] Golsteijn L, Menkveld R, King H, Schneider C, Schowanek D, Nissen SA (2015). Compilation of life cycle studies for six household detergent product categories in Europe: the basis for product-specific A.I.S.E. Charter Advanced Sustainability Profiles. Environ Sci Eur.

[CR15] Gómez E, González G (2015) Comportamiento del aceite de palma de Colombia en los principales mercados de exportación. Revista Lebret, (7):283–305. https://doi.org/10.15332/rl.v0i7.1528

[CR16] Grigore ME (2017). Methods of recycling, properties and applications of recycled thermoplastic polymers. Recycling.

[CR17] Herrera Torreblanca XJ (2017) Determinación y evaluación comparativa de la cinética de biodegradación de los tensioactivos lauril éter sulfato de sodio (Aniónico), alcohol etoxilado (No Iónico) y cocoamido propil betaína (Anfótero) en condiciones ambientales. Undergraduate thesis, School of Chemical Engineering, National University of San Agustin, Arequipa

[CR18] Huijbregts MAJ, Steinmann ZJN, Elshout PMF, Stam G, Verones F, Vieira MDM, Hollander A, Zijp M, van Zelm R (2017) ReCiPe 2016 v1.1. A harmonized life cycle impact assessment method at midpoint and endpoint level Report I: Characterization, Bilthoven

[CR19] ISO. ISO 14040 (2006) Environmental management — life cycle assessment — principles and framework. vol. 2006. Second edi. Geneva: ISO; 2006

[CR20] Javed A, Qazi JI (2016). Skin health implications of chemical detergents and importance of biodetergents. Punjab Univ J Zool.

[CR21] Kjellin M, Johansson I (2010) Surfactants from renewable resources. John Wiley & Sons, New Jersey

[CR22] Latan H, Chiappetta Jabbour CJ, de Sousa L, Jabbour AB, Wamba SF, Shahbaz M (2018). Effects of environmental strategy, environmental uncertainty and top management’s commitment on corporate environmental performance: the role of environmental management accounting. J Clean Prod.

[CR23] Madariaga BM, Ramos MJ, Tarazona JV (2007) Development of an European quantitative eutrophication risk assessment of polyphosphates in detergents: Model implementation and quantification of the eutrophication risk associated to the use of phosphates in detergents. Final study report. Green Planet Research Report GPR-CEEP-06-2-Final. 2006. Carried out by Green Planet Research and INIA (Spanish National Institute for Agricultural and Food Research and Technology) for CEEP. Published by the EU Commission

[CR24] Mousavi SA, Khodadoost F (2019). Effects of detergents on natural ecosystems and wastewater treatment processes: a review. Environ Sci Pollut Res.

[CR25] Nasution M, Wibawa D, Ahamed T, Noguchi R (2018). Comparative environmental impact evaluation of palm oil mill effluent treatment using a life cycle assessment approach: a case study based on composting and a combination for biogas technologies in North Sumatera of Indonesia. J Clean Prod.

[CR26] Osorio-Tejada JL, Llera-Sastresa E, Hashim AH (2018). Well-to-wheels approach for the environmental impact assessment of road freight services. Sustainability.

[CR27] Osorio-Tejada J, Tran NN, Hessel V (2022) Techno-environmental assessment of small-scale Haber-Bosch and plasma-assisted ammonia supply chains. Sci Total Environ 826:154162. 10.1016/j.scitotenv.2022.154162 10.1016/j.scitotenv.2022.154162 35240177

[CR28] Parlour L (2017) Palm oil effects and soaps that contain pal oil. https://levityparlour.com/blogs/news/palm-oil-effects-and-soaps-that-contain-palm-oil. Accessed 7 Dec 2022

[CR29] Pertuz Martínez AP, Santamaría Escobar ÁE (2014). La Palmicultura Colombiana: Sostenibilidad Económica, Social Y Ambiental. Tendencias.

[CR30] PRé Consultants (2022) SimaPro v9. About SimaPro 2022. www.pre.nl/content/simapro-lca-software (accessed January 16, 2022)

[CR31] Rebello S, Anoopkumar AN, Sindhu R, Binod P, Pandey A, Aneesh EM (2019) Comparative life-cycle analysis of synthetic detergents and biosurfactants-an overview, refining biomass residues for sustainable energy and bioproducts: technology, advances, life cycle assessment, and economics. Elsevier Inc. 10.1016/B978-0-12-818996-2.00023-5

[CR32] Ríos LA, Franco A, Echeverri DA (2006). Producción de alcoholes grasos a partir de aceites de palma y de palmiste utilizando procesos de hidrogenación catalítica. Una Revisión Bibliográfica Palmas.

[CR33] Saswattecha K, Kroeze C, Jawjit W, Hein L (2015). Assessing the environmental impact of palm oil produced in Thailand. J Clean Prod.

[CR34] Saxena N, Pal N, Dey S, Mandal A (2017). Characterizations of surfactant synthesized from palm oil and its application in enhanced oil recovery. J Taiwan Inst Chem Eng.

[CR35] Schmidt JH (2010). Comparative life cycle assessment of rapeseed oil and palm oil. Int J Life Cycle Assess.

[CR36] Singh P, Patil Y, Rale V (2019). Biosurfactant production: emerging trends and promising strategies. J Appl Microbiol.

[CR37] Slamet, Ibadurrohman M, Wulandari PP (2017) Synthesis of methyl ester sulfonate surfactant from crude palm oil as an active substance of laundry liquid detergent. AIP Conf Proc 1904. 10.1063/1.5011915

[CR38] Smulders E, von Rybinski W, Nordskog A (2012) Laundry detergents, 1. Introduction. In: Ullmann’s Encyclopedia of Industrial Chemistry. Wiley-VCH Verlag GmbH & Co., KGaA, Weinheim

[CR39] Solé Cabanes A (2014). Tensioactivos en la industria textil. 3C Tecnol.

[CR40] Vallejo AMA (2004) Utilización del Análisis del ciclo de vida en la evaluación del impacto ambiental del cultivo bajo invernadero mediterráneo. PhD thesis, Departament de Projectes d'Enginyeria, Universitat Politècnica de Catalunya, Barcelona

[CR41] Viva Q (2013). Producción de plástico parcialmente degradable con polietileno de alta densidad (PEAD) y la dextrina del desecho de Solanum tuberosum. Química Viva.

[CR42] Wang M, Tan G, Eljaszewicz A, Meng Y, Wawrzyniak P, Acharya S, Altunbulakli C, Westermann P, Dreher A, Yan L, Wang C, Akdis M, Zhang L, Nadeau KC, Akdis CA (2019). Laundry detergents and detergent residue after rinsing directly disrupt tight junction barrier integrity in human bronchial epithelial cells. J Allergy Clin Immunol.

[CR43] Zhao X, Cornish K, Vodovotz Y (2020). Narrowing the gap for bioplastic use in food packaging: an update. Environ Sci Technol.

