# Supplementary material for: Comparative life cycle assessment for the manufacture of bio-detergents
Source: Environ Sci Pollut Res Int. 2022 Dec 12;30(12):34243–54. doi: 10.1007/s11356-022-24439-x (PMC10017589; doi:10.1007/s11356-022-24439-x)
Supplement: Supplementary file 1 — Supplementary file1 (DOCX 30.9 KB) [file 11356_2022_24439_MOESM1_ESM.docx]

SUPPLEMENTARY MATERIAL (SM1) FOR THE ARTICLE:

**Comparative life cycle assessment for the manufacture of bio-detergents**

Javier Mauricio Villota-Paz ^ab^, José Luis Osorio-Tejada ^ac^ ^[[1]](#footnote-1)^*, Tito Morales-Pinzón ^a^

^a^ *Faculty of Environmental Sciences, Universidad Tecnológica de Pereira, Alamos, Pereira, Colombia.*

*^b^Faculty of Engineering, Universidad Mariana, Pasto, Colombia.*

^c^ *School of Engineering, University of Warwick, Coventry, United Kingdom.*

**Contents**

**Table S1** *LCI one liter of Bio-detergent (Petrochemical and PET container) BLD*

**Table S2** *LCI one liter of Bio-detergent (Petrochemical and HDPE container) BLD*

**Table S3** *LCI one liter Bio-detergent (30% Vegetable surfactant and 70% petrochemical) BLD*

**Table S4** *LCI one kilogram of Solid Detergent TD*

**Table S1**

*LCI one liter of Bio-detergent (Petrochemical and PET container) BLD*

| **Raw materials** | **Selected material dataset** | **Provider location** | **Distance**  **(km)** | **tkm** | **Amount** | **Unit** |
| --- | --- | --- | --- | --- | --- | --- |
| Fatty alcohol sulfates | Fatty alcohol sulfate {RoW}\| production, petrochemical \| Cut-off, S | Cra. 34 #13-150, Yumbo Valle del Cauca | 392 | 0.03724 | 0.095 | kg |
| Sodium Chloride | Sodium chloride, powder {RoW}\| production \| Cut-off, S | Calle 20 #21B - 16, Pasto, Nariño | 14.4 | 0.0008208 | 0.057 | kg |
| PET container | Polyethylene terephthalate, granulate, bottle grade {RoW}\| production \| Cut-off, S | Ak 30 #20, Bogotá, Cundinamarca | 936 | 0.00936 | 0.01 | kg |
| Labels | Printed paper, offset {RoW}\| offset printing, per kg printed paper \| Cut-off, S | Cl. 17 #23-84, Pasto, Nariño | 14.7 | 0.00001764 | 0.0012 | kg |
| Cardboard boxes | Corrugated board box {RoW}\| production \| Cut-off, S | Alto de Daza | 7.9 | 0.0000948 | 0.012 | kg |
| Energy consumption | Electricity, medium voltage {CO}\| electricity voltage transformation from high to medium voltage \| Cut-off, S | Cedenar S.A E.S.P | N/A | N/A | 0.0392 | MJ |
| Water consumption | Tap water {CO}\| tap water production, direct filtration treatment \| Cut-off, S | Quebrada Meneses | N/A | N/A | 1.73 | kg |
| Distribution | Transport, freight, lorry, unspecified {RoW}\| transport, freight, lorry, all sizes, EURO4 to generic market for \| Cut-off, S | Alto de Daza, Cra. 11 #15-17, Pasto, Nariño B/ las Violetas, Cl. 20a #2a16, Pasto, Nariño B/Las mercedes | 38.7 | 0.039861 | 0.04 | tkm |

**Table S2**

*LCI one liter of Bio-detergent (Petrochemical and HDPE container) BLD*

| **Raw materials** | **Selected dataset** | **Provider location** | **Distance**  **(km)** | **tkm** | **Amount** | **Unit** |
| --- | --- | --- | --- | --- | --- | --- |
| Fatty alcohol sulfates | Fatty alcohol sulfate {RoW}\| production, petrochemical \| Cut-off, S | Cra. 34 #13-150, Yumbo Valle del Cauca | 392 | 0.03724 | 0.095 | kg |
| Sodium Chloride | Sodium chloride, powder {RoW}\| production \| Cut-off, S | Calle 20 #21B - 16, Pasto, Nariño | 14.4 | 0.0008208 | 0.057 | kg |
| HDPE container | Polyethylene, high density, granulate {RoW}\| production \| Cut-off, S | Ak 30 #20, Bogotá, Cundinamarca | 936 | 0.020592 | 0.022 | kg |
| Labels | Printed paper, offset {RoW}\| offset printing, per kg printed paper \| Cut-off, S | Cl. 17 #23-84, Pasto, Nariño | 14.7 | 0.00001764 | 0.0012 | kg |
| Cardboard boxes | Corrugated board box {RoW}\| production \| Cut-off, S | Alto de Daza | 7.9 | 0.0000948 | 0.012 | kg |
| Energy consumption | Electricity, medium voltage {CO}\| electricity voltage transformation from high to medium voltage \| Cut-off, S | Cedenar ESP | N/A | N/A | 0.0392 | MJ |
| Water consumption | Tap water {CO}\| tap water production, direct filtration treatment \| Cut-off, S | Quebrada Menses | N/A | N/A | 1.73 | kg |
| Distribution | Transport, freight, lorry, unspecified {RoW}\| transport, freight, lorry, all sizes, EURO4 to generic market for \| Cut-off, S | Alto de Daza, Cra. 11 #15-17, Pasto, Nariño B/ las Violetas, Cl. 20a #2a16, Pasto, Nariño B/Las mercedes | 38.7 | 0.039861 | 0.04 | tkm |

**Table S3**

*LCI one liter Bio-detergent (30% Vegetable surfactant and 70% petrochemical) BLD*

| **Raw materials** | **Selected material dataset** | **Provider location** | **Distance**  **(km)** | **tkm** | **Amount** | **Unit** |
| --- | --- | --- | --- | --- | --- | --- |
| Fatty alcohol sulfates | Fatty alcohol sulfate {RoW}\| production, petrochemical \| Cut-off, S | Cra. 34 #13-150, Yumbo Valle del Cauca | 392 | 0.026068 | 0.0665 | kg |
| Fatty alcohol sulfate, palm oil | Fatty alcohol sulfate {RoW}\| production, palm oil \| Cut-off, S | Cra. 34 #13-150, Yumbo Valle del Cauca | 392 | 0.011172 | 0.0285 | kg |
| Sodium Chloride | Sodium chloride, powder {RoW}\| production \| Cut-off, S | Calle 20 #21B - 16, Pasto, Nariño | 14.4 | 0.0008208 | 0.057 | kg |
| PET container | Polyethylene terephthalate, granulate, bottle grade {RoW}\| production \| Cut-off, S | Ak 30 #20, Bogotá, Cundinamarca | 936 | 0.00936 | 0.01 | kg |
| Labels | Printed paper, offset {RoW}\| offset printing, per kg printed paper \| Cut-off, S | Cl. 17 #23-84, Pasto, Nariño | 14.7 | 0.00001764 | 0.0012 | kg |
| Cardboard boxes | Corrugated board box {RoW}\| production \| Cut-off, S | Alto de Daza | 7.9 | 0.0000948 | 0.012 | kg |
| Energy consumption | Electricity, medium voltage {CO}\| electricity voltage transformation from high to medium voltage \| Cut-off, S | Cedenar ESP | N/A | N/A | 0.0392 | MJ |
| Water consumption | Tap water {CO}\| tap water production, direct filtration treatment \| Cut-off, S | Quebrada Menses | N/A | N/A | 1.73 | kg |
| Distribution | Transport, freight, lorry, unspecified {RoW}\| transport, freight, lorry, all sizes, EURO4 to generic market for \| Cut-off, S | Alto de daza, Cra. 11 #15-17, Pasto, Nariño B/ las Violetas, Cl. 20a #2a16, Pasto, Nariño B/Las mercedes | 38.7 | 0.039861 | 0.04 | tkm |

Minor ingredients such as preservatives and isothiazolinone copper salts, which are present in very low concentrations (~0.01%), were omitted.

**Table S4**

*LCI one kilogram of Solid Detergent TD*

| **Raw materials** | **Selected dataset** | **Provider location** | **Distance**  **(km)** | **tkm** | **Amount** | **Unit** |
| --- | --- | --- | --- | --- | --- | --- |
| Sodium Hydroxide | Sodium hydroxide, without water, in 50% solution state {RoW}\| chlor-alkali electrolysis, diaphragm cell \| Cut-off, S | Cra 64 #5a - 97 Bogotá Colombia | 779 | 0.065436 | 0.084 | Kg |
| Palm oil | Palm oil, refined {GLO}\| palm oil refinery operation \| Cut-off, S | Cra 64 #5a - 97 Bogotá Colombia | 779 | 0.354445 | 0.455 | Kg |
| Water consumption | Tap water {CO}\| tap water production, direct filtration treatment \| Cut-off, S | Quebrada Menses | N/A | N/A | 3.5 | Kg |
| Energy consumption | Electricity, medium voltage {CO}\| electricity voltage transformation from high to medium voltage \| Cut-off, S | Cedenar ESP | N/A | N/A | 0.0392 | MJ |
| Distribution | Transport, freight, lorry, unspecified {RoW}\| transport, freight, lorry, all sizes, EURO4 to generic market for \| Cut-off, S | Alto de daza, Cra. 11 #15-17, Pasto, Nariño B/ las Violetas, Cl. 20a #2a16, Pasto, Nariño B/Las mercedes | 38.7 | 0.039861 | 0.04 | tkm |

1. ** Correspondence author.*

   *E-mail*: [jose.osorio-tejada@warwick.ac.uk](mailto:javier.villota@utp.edu.co) (J. Osorio-Tejada)

   *Phone*: +44 7727959525 [↑](#footnote-ref-1)
