# Supplementary material for: Comparative life cycle assessment for the manufacture of bio-detergents
Source: Environ Sci Pollut Res Int. 2022 Dec 12;30(12):34243–54. doi: 10.1007/s11356-022-24439-x (PMC10017589; doi:10.1007/s11356-022-24439-x)
Supplement: Supplementary file 2 — Supplementary file2 (PDF 788 KB) [file 11356_2022_24439_MOESM2_ESM.pdf]

SUPPLEMENTARY MATERIAL (SM2) FOR THE ARTICLE:

Comparative life cycle assessment for the manufacture of bio-detergents

Javier Mauricio Villota-Paz [ab], José Luis Osorio-Tejada [ac] *, Tito Morales-Pinzón [a]

[a] *Faculty of Environmental Sciences, Universidad Tecnológica de Pereira, Alamos, Pereira, Colombia.*
[b]*Faculty of Engineering, Universidad Mariana, Pasto, Colombia.*
[c] *School of Engineering, University of Warwick, Coventry, United Kingdom.*

Contents

Technical datasheet biodegradability test for the plant-based Biomultipurpose liquid detergent

* *Correspondence author.*
E-mail: jose.osorio-tejada@warwick.ac.uk (J. Osorio-Tejada)
Phone: +44 7727959525

TECNIMICRO
Laboratorio de Análisis S.A.S.

SUPLEMENTO AL/SUMPLEMENT TO
REPORTE DE ANÁLISIS/ANALYSIS REPORT

Muestra/Sample #: 279993

Empresa / Company: **PROTECNICA INGENIERIA S.A.S** **Telf. / Phone:** (2) 444 51 97- 69

Sede / Headquarters: **Sede principal** Carrera 34 No. 13-150 Arroyohondo Yumbo - Cali

Producto / Product: **PROBLEND DT 612**

Muestra / Sample: Sin identificación

Fecha de Recepción / Reception date: 28/06/2018 **Temp. de Recepción / Temp. Reception:** 23,7°C

Cantidad / Amount: 500 ml

Contenedor / Container: Recipiente plástico con tapa/Plastic container with lid

Enviada por /Send by: Johan Sebastián Londoño

Aspecto / Aspect: Líquido ligeramente viscoso, homogéneo, libre de partículas/ Liquid slightly viscous, homogeneous, free of particles

Olor / Smell: Característico del producto / Characteristic of the product

Color / Color: Incoloro / Colorless.

Análisis Fisicoquímico/ Physicochemical Analysis	Fecha/ Date	Unidad/ Unit	Resultado/ Result	Especificaciones(**)/ Specifications(**)	Método/ Method
Biodegradabilidad en el punto final/Biodegradability at the final point	21/09/2018	%	66,35± 2,64	>60	OCDE 301 F. Respirometría manométrica*
DBO/DBO	21/09/2018	mg O2/L	56,4	56	OCDE 301 F (Respirometría manométrica)
DQO/DQO	21/09/2018	mg O2/L	DQO Inicial:(1028); DQO Final:(85)	NA	OCDE 301 F (Respirometría manométrica)

COPIA DIGITAL

Tecnimicro Laboratorio de Análisis S.A.S.
Carrera 42 Nº 10-37 Medellín
Colombia
PBX (4) 4031100 / FAX (4) 268 1996
www.tecnimicro.net

Una empresa del grupo
MERIEUX NutriSciences

(**) Según Norma / Acoordinng to Standard:

(*) Métodos acreditados/Methods Accredited: ONAC, Certificado/Certificate: 10-LAB-053 Fecha/Date: 2014-09-05 ISO/IEC 17025:2005

NOTA: Este resultado corresponde, exclusivamente a la muestra recibida y analizada en el laboratorio/This result correspond only to the sample received and analyzed by the laboratory

La muestra recibida y analizada en el laboratorio, cumple con el parámetro de biodegradabilidad, evaluado según especificación establecida por la NTC 5604 y la RESOLUCIÓN CONJUNTA N° 1770, Ministerio de Salud y Protección Social - Ministerio de Ambiente y Desarrollo Sostenible, ensayo realizado en el tensoactivo extraído de la muestra según la END 0065, equivalente al Reglamento (CE) No 648/2004 del Parlamento Europeo y del Consejo de 31 de marzo de 2004 sobre detergentes, con un nivel de confianza del 95% y un K=2 / The sample received and analyzed in the laboratory, meets the parameter of biodegradability evaluated according to specification established by NTC 5604 and JOINT RESOLUTION No. 0689, Ministry of

Juan G. Higuita Pérez
T.P.01533343103062261
Químico Farmacéutico U de A
Coordinador/Coordinator

Fecha de Impresión/printing date: 21/09/2018

Fecha / Date: 21/09/2018

Informe firmado digitalmente, Sin sello seco de TECNIMICRO la información no tiene validez/Digitally signed report, without dry seal of TECNIMICRO this information is not valid

Este informe no puede ser reproducido total ni parcialmente sin autorización de Tecnimicro Laboratorio de Análisis S.A.S/ / This report can't be reproduced in whole or in part without Tecnimicro Laboratorio de Análisis's authorization

Por solicitud del cliente, este informe reemplaza al control/By client's request, this report replaces the sample number: **271555**

FIN DE INFORME/ END OF REPORT

COPIA DIGITAL

Tecnimicro Laboratorio de Análisis S.A.S.
Carrera 42 Nº 10-37 Medellín
Colombia
PBX (4) 4031100 / FAX (4) 268 1996
www.tecnimicro.net

Una empresa del grupo
MERIEUX NutriSciences
